# From scanner to scalpel: adapting IDEAL for accountable AI in radiology and image-guided neurosurgery

**DOI:** 10.3389/fradi.2026.1979076

**Published:** 2026-09-24

**Authors:** Jasleen Saini, Sunam Jassar, Scott J. Adams, Saira B. Alli, Errol Colak, Jefferson R. Wilson

**Affiliations:** 1 Division of Neurosurgery, Department of Surgery, University of Toronto, Toronto, ON, Canada; 2 Li Ka Shing Knowledge Institute, St. Michael’s Hospital, Toronto, ON, Canada; 3 Institute of Health Policy, Management, and Evaluation, University of Toronto, Toronto, ON, Canada; 4 Department of Medical Imaging, University of Toronto, Toronto, ON, Canada; 5 Department of Medical Imaging, University of Saskatchewan, Saskatoon, SK, Canada; 6 Division of Neurosurgery, Department of Surgery, Sunnybrook Health Sciences Centre, Toronto, ON, Canada

**Keywords:** artificial intelligence, clinical governance, neurosurgery, patient outcomes, radiology, regulation, validation

## Abstract

Artificial intelligence (AI) is increasingly integrated into clinical decision-making in radiology and image-guided neurosurgery, yet evidence generation, oversight, and accountability remain uneven across the technology lifecycle. Strong performance on curated test sets may not persist across institutions, scanners, patient subgroups, or changing clinical workflows. Existing reporting guidance, medical-device standards, regulatory requirements, and machine learning operations (MLOps) address important parts of this problem, but are not designed to serve as a unified, stage-gated clinical-evidence pathway across the full lifecycle. We propose IDEAL-AI, an adaptation of the Idea, Development, Exploration, Assessment, and Long-term study principles for clinical AI. IDEAL-AI organizes evidence generation across five stages and three concurrent domains: validation; governance, regulation, and ethics; and patient and system outcomes. It is intended to complement, not replace, established reporting guidelines, regulatory requirements, facility-level quality programs, and MLOps infrastructure. By separating retrospective model development from prospective live exploration, requiring prespecified technical, fairness, human-factors, and clinical endpoints, and extending evaluation through post-market surveillance, the framework offers a practical way to determine when an AI system is ready to progress. Success should be judged not by adoption speed or benchmark accuracy alone, but by reproducible clinical benefit for patients, clinicians, and health systems.

## 1 Introduction

Artificial intelligence (AI) has shifted from a research concept to a defining force in clinical practice. In radiology, algorithms delineate tumor margins, classify molecular signatures, and forecast outcomes before a single incision is made. In neurosurgery, AI may support preoperative planning, image registration, augmented-reality overlays, intraoperative navigation, real-time scene interpretation, and video-based skill assessment (1–4). These surgical applications introduce risks not captured by image-only benchmarks, including registration error, anatomical deformation such as brain shift, latency, device interoperability, operator learning curves, and the consequences of erroneous guidance in real time. Radiology and image-guided neurosurgery therefore provide complementary settings in which to evaluate whether technical performance translates into safe clinical benefit.

Despite remarkable technical progress, the supporting infrastructure for evidence, ethics, and accountability remains incomplete. Models that perform strongly in controlled studies may show reduced performance across new institutions, scanners, workflows, or patient populations (5–8). Scanner calibration, institutional heterogeneity, and underrepresentation of vulnerable populations continue to limit generalizability (6, 7).

To address this gap, we propose the IDEAL-AI framework, an evolution of established surgical innovation principles, to guide the safe, transparent, and accountable integration of AI into clinical practice. The task ahead is clear: to match the pace of innovation with equally rigorous systems of validation and governance (9), and to embed accountability as a core requirement rather than a retrospective consideration.

## 2 From accuracy to accountability

The integration of AI into radiology and neurosurgery has moved beyond proof of capability to questions of consequence. Algorithms can influence when to operate, what tissue to target, and how risk is communicated to patients (10). This integration of machine-assisted judgment demands a parallel evolution in how clinicians define and measure accountability.

Single aggregate measures reported on curated test sets do not, by themselves, characterize how an algorithm behaves under the variability of clinical practice (5). This is not a limitation of metrics in general: prespecified measures of subgroup performance, calibration, uncertainty, robustness, and site-specific or temporal distribution shift can quantify clinically important failure patterns. Accuracy in one curated dataset nevertheless does not guarantee reliability across scanners, institutions, or patient populations, and subtle differences in acquisition or labeling can produce clinically significant errors (8). The central question is whether performance is reproducible, whether uncertainty is calibrated, and whether clinicians and monitoring systems can recognize when performance has degraded (8, 11–13).

Automation bias compounds the challenge, particularly in high-stakes settings such as neurosurgical navigation, where false precision can lead to irreversible harm. It is not simply a transparency problem. Confidence estimates and explanations may help users calibrate reliance, but poorly calibrated confidence, cognitively burdensome explanations, or overly simple rationales may instead reinforce misplaced trust (14, 15). Mitigation therefore requires human-factors design and prospective evaluation, including clinician training and AI literacy, independent review before exposure to AI advice where feasible, usable uncertainty displays, clear escalation and override pathways, and monitoring of disagreement, verification, and override behavior. Systems should actively support, rather than merely permit, clinician challenge (14–16).

Accountability must extend beyond performance to provenance. Every algorithm represents a chain of decisions about data sources, preprocessing, labeling, and optimization that shape its outputs. Transparent lifecycle documentation, change control, and independent external validation are formalized in medical-device software standards and high-risk AI regulation; IDEAL-AI is not intended to duplicate those requirements. Its role is to connect technical documentation and validation to stage-specific clinical evidence and explicit progression criteria (9, 17, 18). Performance audits should be routine rather than exceptional, with findings communicated to regulators, clinicians, institutions, and patients in an appropriate form (6, 10).

Ultimately, accuracy without accountability is an incomplete form of precision. The next phase of AI integration must focus less on outperforming humans and more on empowering them, ensuring that technology strengthens rather than supplants the clinical reasoning at the center of patient care (7, 19).

## 3 Regulation, standards, and accountability in practice

The rapid expansion of AI has exposed a gap between product authorization and local clinical readiness. These challenges are particularly visible in data-intensive and high-stakes fields such as radiology and image-guided surgery. Medical-device regulation and software-lifecycle standards establish essential controls, but adaptive or frequently updated systems also require prospective change management, recalibration criteria, and post-deployment evidence. Jurisdictional approaches remain heterogeneous (20, 21). In the United States, Predetermined Change Control Plans (PCCPs) allow manufacturers to prospectively specify certain modifications and the methods used to develop, validate, and implement them while maintaining regulatory oversight (22, 23).

In many health systems, healthcare AI governance remains fragmented across institutions, with adoption shaped by local procurement and key decisions about validation, safety, and oversight left to individual organizations (24). When errors occur, responsibility may be distributed among clinicians, institutions, vendors, and developers (25). Professional responsibility does not disappear when AI is used, but probabilistic outputs and opaque development processes can complicate informed consent, documentation, and explanation (9, 20). Governance should therefore assign responsibilities across the lifecycle rather than assume that the clinician's ability to override a recommendation is, by itself, an adequate safeguard.

A lifecycle governance model offers one path forward. Due diligence should include clear audit trails, local acceptance testing, post-market monitoring, calibration review, and structured management of model updates. Hospitals can support this process through multidisciplinary Clinical AI Oversight Boards that include clinicians, data scientists, information-technology and quality leaders, ethicists, patient representatives, and legal or regulatory experts. Such boards should define accountability, escalation pathways, stop rules, and the evidence required before deployment or expansion (10, 25).

Linked health-data systems, including national or regional registries, can enable real-world performance tracking across institutions and populations (26). Federated evaluation can extend this model through harmonized local analyses and aggregated results without centralizing patient-level data (27). Federation still requires common definitions, label-quality assurance, interoperability, privacy review, and auditable governance. This turns oversight from a checkpoint into an ongoing partnership among developers, clinicians, institutions, patients, and regulators (25, 28).

## 4 The IDEAL-AI framework: lessons from surgical innovation

Surgical innovation has long grappled with the tension between discovery and safety. The IDEAL framework, which stands for Idea, Development, Exploration, Assessment, and Long-term study, was originally designed to standardize the evaluation of surgical and interventional innovations (28). IDEAL-AI adopts this stage logic while accounting for a central difference: an algorithm may be trained on thousands or millions of retrospective observations yet still have no evidence from live clinical use. Maturity should therefore not be inferred from a fixed patient-count range. Stage assignment should reflect intended use, risk, site and scanner diversity, outcome prevalence, estimation precision, subgroup power, model stability, and whether evaluation is retrospective, silent prospective, or live. Three concurrent domains, including validation; governance, regulation, and ethics; and patient and system outcomes, span every stage (Figure 1).

**Figure 1:**
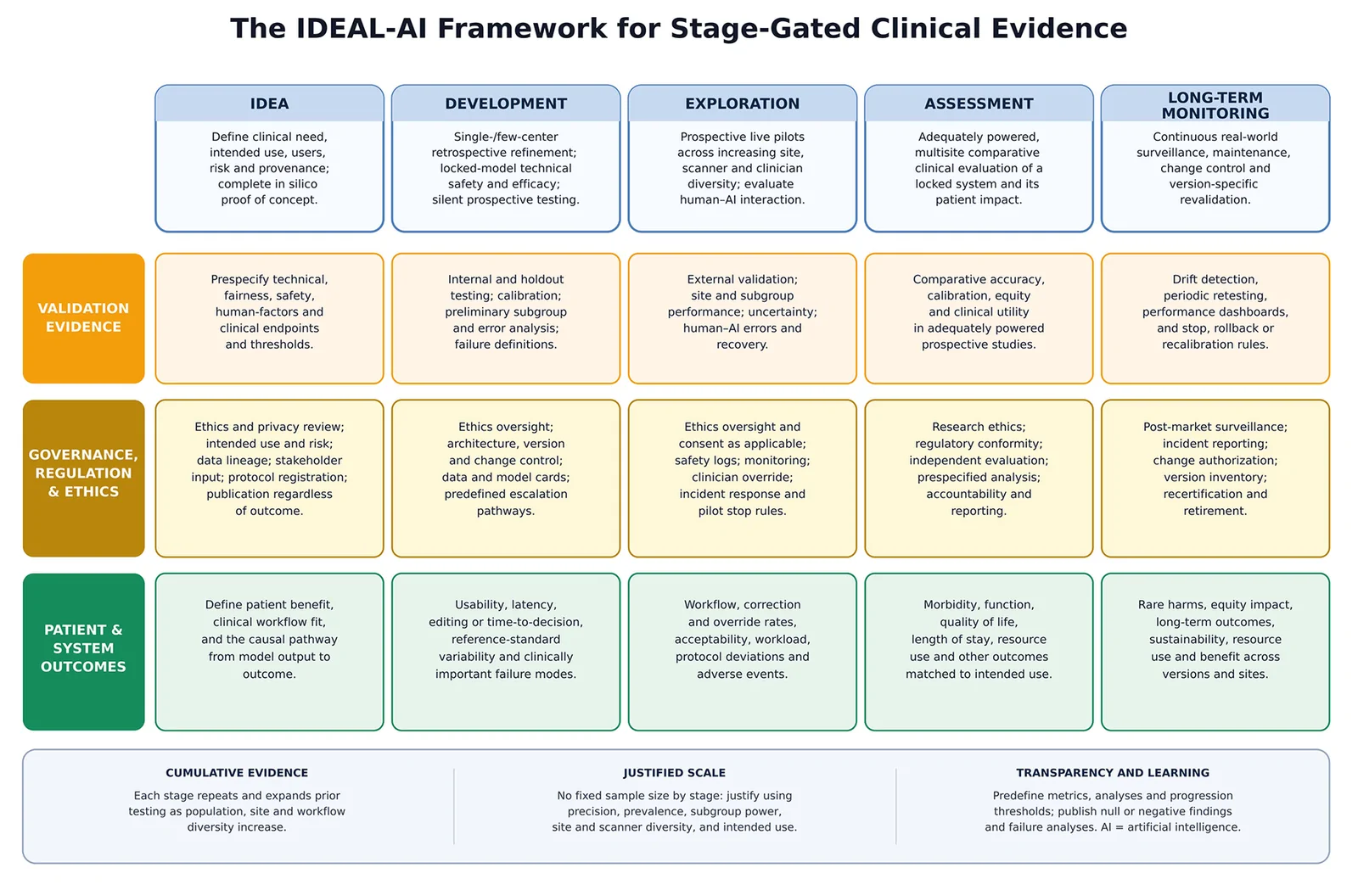
The IDEAL-AI framework for stage-gated evidence. Five stages map progression from a defined clinical need and in silico proof of concept to retrospective development, prospective live exploration, comparative assessment, and long-term monitoring. Validation; governance, regulation, and ethics; and patient and system outcomes are evaluated at every stage, with tests repeated and expanded as site and population diversity increases. Sample size is not fixed by stage and should be justified by intended use, event prevalence, estimation precision, subgroup power, and site or scanner diversity. AI, artificial intelligence.

At the Idea stage, AI development should begin with a defined clinical need, intended use, users, risk class, data provenance, and causal pathway from model output to patient benefit, rather than with a technical challenge in search of an application (16, 28–30). In silico proof of concept belongs in this stage. Technical, fairness, safety, human-factors, and clinical endpoints, including thresholds for progression or stopping, should be specified before model tuning, and protocols should be registered when feasible. Negative or null results and failure analyses should be made available so that ineffective approaches are not repeatedly pursued (28, 30).

The Development and Exploration stages should remain distinct. Development (IDEAL stage 2a) covers model refinement and initial technical safety and efficacy, usually in a single or small number of centers and often using retrospective data. It requires separated training, tuning, and locked test sets; version and change control; prespecified error and subgroup analyses; and, before live use, silent prospective evaluation where feasible. Early bias analyses at this stage are necessarily preliminary and must be repeated as diversity increases. Exploration (stage 2b) begins prospective live clinical use across increasing site and clinician diversity. Its focus is generalizability, workflow integration, usability, human–AI interaction, error recovery, and safety under real conditions. This separation prevents broad clinical claims from being inferred from retrospective development alone (3, 9, 19, 25, 31, 32).

The Assessment phase requires adequately powered, multi-institutional comparative evaluation of a locked system, with a prospectively specified protocol and analysis plan (28). Randomization is preferred when feasible; justified alternatives should address secular trends, selection, learning curves, and site effects. This phase is conceptually aligned with the Fryback and Thornbury hierarchy, which progresses from technical performance to clinical and societal efficacy (33). Evaluation should connect model outputs to clinician behavior, workflow, safety, equity, patient-reported outcomes, morbidity, recovery, and resource use, rather than assuming that an improvement in accuracy will produce clinical benefit (5, 10, 31, 32).

The Long-term phase ensures that oversight continues beyond authorization or local deployment (28, 29). Machine learning operations (MLOps) provide the operational infrastructure for versioning, data and model monitoring, reproducible deployment, audit trails, and controlled updates in healthcare (11–13). Hospitals should use real-world dashboards to track calibration, subgroup performance, drift, safety signals, and the relationship between predictions, clinician actions, and outcomes over time (26). Predefined alert, stop, rollback, recalibration, and revalidation rules should be linked to change-control processes such as PCCPs when applicable (9, 23, 25).

IDEAL-AI links regulatory clearance, standards conformity, reporting guidance, procurement safeguards, and MLOps to explicit clinical-evidence questions and progression decisions. A technically compliant system may still lack evidence of workflow fit or patient benefit, while a clinically promising pilot may remain unready for scale because of inadequate documentation, subgroup evaluation, or change control. The framework therefore treats validation as cumulative evidence rather than a one-time checkpoint (25, 29).

### 4.1 Relationship to existing frameworks

IDEAL-AI is designed as an orchestration layer rather than a competitor to existing radiology, reporting, regulatory, or operational frameworks. Table 1 summarizes the principal areas of overlap and distinction. Facility programs and consensus statements guide procurement and deployment; registries and MLOps provide operational monitoring; software toolkits support implementation; and reporting guidelines improve transparency. IDEAL-AI adds a cross-specialty sequence of evidence questions, stage-transition criteria, and patient and system endpoints from proof of concept through long-term use.

**Table 1:** Summary of existing clinical AI frameworks and resources and their relationship to IDEAL-AI.

Framework or resource	Primary contribution	Relationship to IDEAL-AI
ARCH-AI (34)	Facility-level governance, inventory, local testing, workflow integration, and ongoing quality assurance in radiology.	IDEAL-AI begins before procurement or deployment, links evidence to stage-transition decisions, and extends the logic to image-guided surgery and patient outcomes.
Assess-AI (26)	Registry infrastructure for post-deployment monitoring, benchmarking, and review of discordant imaging-AI cases.	Operationalizes part of Long-term monitoring; IDEAL-AI also specifies predeployment Development, prospective Exploration, comparative Assessment, and outcome pathways.
Multi-society radiology statement (35)	Practical ethical and safety considerations for developing, purchasing, implementing, and monitoring radiology AI.	IDEAL-AI organizes these considerations temporally and makes progression, stopping, and evidence-expansion decisions explicit.
RSNA–MICCAI perspectives (36)	Clinical, cultural, computational, and regulatory considerations for successful radiology-AI deployment.	IDEAL-AI converts these cross-cutting considerations into cumulative requirements at defined evidence stages and adds surgical human-factors concerns.
CyclOps (37)	Software toolkit and cyclical workflow for developing and operationalizing machine-learning models for healthcare.	Provides technical implementation support; IDEAL-AI is an evaluative and governance pathway centered on clinical evidence and patient benefit.
Resilience-aware MLOps (38)	Technical resilience to drift, out-of-distribution data, adversarial or fault disturbances, uncertainty, and graceful degradation.	Supplies methods for Validation and Long-term monitoring; IDEAL-AI additionally requires live clinical evaluation, ethics, comparative utility, and outcomes.
Clinical Deployment Environments (11)	Institutional infrastructure for real-world development, MLOps, responsible AI, implementation science, and continuous evaluation.	Provides the environment in which IDEAL-AI studies can occur; IDEAL-AI specifies the maturity questions and evidence needed to move between stages.
MLHOps/MedMLOps (12, 13)	Operational pipelines for reproducible deployment, monitoring, maintenance, updating, data protection, and usability in healthcare and radiology.	Serve as the operational substrate; IDEAL-AI adds stage-gated clinical evidence, prospective human-factors evaluation, and explicit patient-centered progression criteria.
DECIDE-AI (16)	Reporting guideline for early live clinical evaluation of AI decision-support systems.	Directly complements Development and Exploration; IDEAL-AI spans the full lifecycle and is an evaluation pathway rather than a reporting checklist.

The 2019 update of IDEAL added a preclinical stage, reinforced registries, required ethical attention throughout the lifecycle, and emphasized reporting regardless of outcome (30). IDEAL-AI incorporates these principles while accommodating retrospective data for model training and refinement during *Development*. Prospective evaluation becomes necessary before and during live *Exploration*, and later *Assessment* should use adequately powered comparative designs. Sample size is not assigned by a fixed range; it must be justified by intended use, precision, prevalence, subgroup power, and site or scanner diversity.

Standards and law have distinct roles. The IEC catalogue lists IEC 62304:2006 + A1:2015 as the current published medical-device software lifecycle standard (17). The European Union Artificial Intelligence Act establishes phased obligations for high-risk AI, including risk management, documentation, logging, human oversight, robustness, and post-market monitoring (18). IDEAL-AI does not add a parallel compliance layer; it organizes the clinical evidence needed to demonstrate whether a compliant system is also safe, useful, equitable, and ready to advance.

### 4.2 Worked example: glioma segmentation

Systematic reviews of glioma-segmentation models report strong Dice similarity coefficients but also frequent reuse of curated public datasets, incomplete reporting, limited external validation, and little prospective clinical evaluation (39, 40). The field therefore often stops at the *Idea* or *Development* stage despite high benchmark performance. For a segmentation tool intended to support glioma surgery, technical overlap is necessary but insufficient: the protocol must define how the contour changes planning, what errors matter near eloquent structures, who reviews and edits the output, and which downstream outcomes are plausibly affected. Table 2 provides an illustrative set of study designs, endpoints, and progression criteria across IDEAL-AI. Endpoint selection should be tailored if the intended use is radiotherapy planning, longitudinal response assessment, or another task.

**Table 2:** Illustrative IDEAL-AI evaluation plan for a glioma-segmentation tool intended to support surgical planning.

Stage	Suggested design and scope	Illustrative endpoints and progression criteria
Idea	Define the intended surgical-planning use, users, risk, data provenance, reference standard, and pathway to patient benefit; prospectively register or publicly archive the protocol and thresholds when feasible.	Prespecify tumor-subregion Dice, 95th-percentile Hausdorff distance, lesion-wise sensitivity, volume error, voxel-wise uncertainty calibration, failure definitions, subgroup variables, and clinically unacceptable boundary errors. Proceed only after an in silico proof of concept and public reporting of failures or null results.
Development	Retrospective single- or few-center development with separated training, tuning, and locked testing; add a silent prospective study before live use where feasible.	Assess inference failures, latency, edit time, inter-rater variability, site and subgroup performance, and errors near eloquent structures. Freeze the model and document changes. Proceed when prespecified technical and safety thresholds are met without an unmitigated catastrophic failure mode.
Exploration	Prospective live pilot across increasing scanner, institution, and clinician diversity with human oversight and staged safety review.	Measure time to an approved contour, correction and override rates, planning changes, workflow latency, usability, clinician workload, confidence calibration, protocol deviations, and adverse events. Conduct semistructured interviews or direct workflow observations with surgeons, radiologists, and technologists, using thematic analysis to assess workflow fit, trust calibration, usability, and failure recovery. Proceed when feasibility, safety, qualitative acceptability, and site or subgroup stability are demonstrated.
Assessment	Adequately powered multisite comparison of AI-assisted and standard planning using a locked version; randomize when feasible and prespecify the analysis.	Evaluate planning time, contour or target agreement, surgical-plan changes, extent of resection, new neurological deficit, quality of life, time to adjuvant therapy, and resource use, selecting outcomes that match the intended causal pathway. Require clinically meaningful benefit without unacceptable inequity or harm.
Long-term monitoring	Registry- and MLOps-supported surveillance across versions, scanners, protocols, sites, and patient populations.	Track calibration and performance drift, subgroup equity, alert burden, rare harmful errors, downstream outcomes, sustainability, and resource use. Predefine alert, stop, rollback, recalibration, and revalidation thresholds; evaluate each major update at the appropriate earlier stage.

## 5 The path forward

Radiology and image-guided neurosurgery occupy the frontier of data-driven care. AI used for diagnosis, risk prediction, planning, navigation, or intraoperative assistance should be evaluated with evidence proportional to its intended use and potential harm (19, 29). IDEAL-AI provides a stage-gated blueprint for that work by linking prespecified metrics, cumulative validation, continuous ethics and governance, comparative clinical evaluation, and post-market monitoring across the lifecycle.

The measure of success will not be how quickly AI is implemented but how rigorously it is evaluated and governed. Clinicians, regulators, and developers share a collective duty to ensure that accuracy, equity, and accountability evolve in tandem. The next era of AI in medicine will be defined not by who adopts it first, but by how effectively it improves patient outcomes.

## Data Availability

The original contributions presented in the study are included in the article/Supplementary Material, further inquiries can be directed to the corresponding author.
